# Isolated orofacial dystonia and facial pain as the inaugural manifestation of anti-NMDAR encephalitis: a case report and literature review

**DOI:** 10.3389/fimmu.2026.1885984

**Published:** 2026-07-30

**Authors:** Youjia Liu, Sihui Chen, Yanyun Wu, Wenting Zhou, Huifang Shang, Xueping Chen, Ruwei Ou

**Affiliations:** Department of Neurology, West China Hospital, Sichuan University, Chengdu, Sichuan, China

**Keywords:** anti-NMDAR encephalitis, autoimmune, case report, isolated orofacial dystonia, movement disorder, temporomandibular joint

## Abstract

Anti-N-methyl-D-aspartate receptor (anti-NMDAR) encephalitis typically presents with psychiatric symptoms, seizures, and cognitive dysfunction. Movement disorders, when present, usually coexist with these features. We report a rare case of anti-NMDAR encephalitis in a 30-year-old female who presented with isolated left temporomandibular joint (TMJ) dystonia, jaw tremor, and facial pain as the sole manifestations for seven months, without clinically significant psychiatric, cognitive, or seizure symptoms. The patient was repeatedly misdiagnosed with TMJ disorder and muscular dysfunction before anti-NMDAR IgG antibodies were detected by cell-based assay (CBA) in serum (1:32) and cerebrospinal fluid (CSF, 1:1). First-line immunotherapy with intravenous immunoglobulin (IVIG) achieved remission. However, she relapsed five months later following COVID-19 infection, with antibody titers rising to 1:100 in both serum and CSF. High-dose corticosteroid pulse therapy and maintenance mycophenolate mofetil were administered, achieving sustained clinical improvement over four years of follow-up, although mild residual focal dystonia persisted. Whole-exome sequencing and hereditary ataxia screening excluded genetic causes. This case broadens the clinical spectrum of anti-NMDAR encephalitis and underscores the importance of early antibody testing in young patients with treatment-refractory orofacial movement disorders.

## 1 Introduction

Anti-N-methyl-D-aspartate receptor (anti-NMDAR) encephalitis is an immune-mediated disorder predominantly affecting young women, characterized by autoantibodies targeting the NR1 subunit of the NMDA receptor (1). The classic presentation follows a multistage progression: prodromal symptoms lead to psychiatric manifestations, seizures, cognitive decline, and movement disorders (2, 3).

Movement disorders occur in 50-80% of patients, with orofacial dyskinesia, chorea, dystonia, and stereotyped movements being the most common phenotypes (4, 5). However, isolated movement disorders without accompanying psychiatric or cognitive symptoms are exceedingly rare. Only a handful of cases have been reported, including isolated hemidystonia (6) and isolated craniocervical dystonia (7).

Isolated TMJ disorders are common conditions attributed to mechanical dysfunction, arthritis, or myofascial pain (8). The misdiagnosis of anti-NMDAR encephalitis presenting as TMJ disorder can lead to prolonged diagnostic delay and poor outcomes. Here, we report the first case of anti-NMDAR encephalitis presenting with isolated TMJ dystonia and facial pain persisting for seven months without neuropsychiatric features, followed by a targeted review of the relevant literature.

## 2 Case description

A previously healthy 30-year-old female presented with sudden-onset left jaw tremor, paroxysmal spasms, involuntary tongue biting, difficulty opening the mouth, and left facial pain in March 2022. She denied psychiatric symptoms, seizures, cognitive impairment, or altered consciousness. There was no relevant family history or recent vaccination.

Over the subsequent seven months, she was repeatedly misdiagnosed. At an oral and maxillofacial surgery department, she was diagnosed with “left TMJ hyperplasia” and received intra-articular injections. At a neurology department, “muscular dysfunction” was diagnosed, and she was treated with tizanidine, trihexyphenidyl, and clonazepam with only mild improvement. Brain MRI was normal. Carbamazepine provided transient relief but caused gastric intolerance(Timeline of clinical events see **Table 1**).

**Table 1:** Timeline of clinical events.

Date	Event
March 2022	Onset of left jaw tremor, spasms, tongue biting, facial pain and swelling
March-Apr 2022	Diagnosed as TMJ hyperplasia; intra-articular lubricant injections (3 weeks)
Apr-Jun 2022	Neurology referral: diagnosed as muscular dysfunction; tizanidine, trihexyphenidyl, clonazepam
July 2022	Symptom worsening; brain MRI normal; medication adjustment ineffective
Aug-Sept 2022	Carbamazepine transient relief; self-discontinued due to gastric discomfort
October 19, 2022	Admission to West China Hospital; comprehensive assessment
October 21, 2022	CSF analysis: anti-NMDAR antibody positive (CSF 1:1, serum 1:32); WES negative
October 25-29, 2022	IVIG treatment (0.4 g/kg/day x 5 days); marked symptom improvement
November 2022	Discharge on prednisone taper; maintenance therapy initiated
March 2023	Relapse following COVID-19 infection; antibody titers elevated (serum 1:100, CSF 1:100)
March 21, 2023	Readmission; IVIG + methylprednisolone pulse (1000 mg x 5 days)
March 2023	Mycophenolate mofetil initiated as maintenance therapy
April 6, 2023	Discharge with marked improvement
2023-2026	Sustained clinical improvement on maintenance immunosuppression with residual focal dystonia
January 2026	Deutetrabenazine (Austedo) 12 mg BID initiated for residual symptoms
April 2026	Latest follow-up; stable on maintenance therapy; botulinum toxin planned

### 2.1 First hospitalization (october 2022)

On admission, neurological examination revealed preserved global cognitive function (MMSE 30/30, MoCA 29/30, FAB 16/18). Formal psychological assessment performed by the Neurofunctional Examination Center and the Clinical Psychology Assessment and Treatment Center demonstrated minimal anxiety and depressive symptoms (HAMA 4, HAMD 2), without evidence of clinically significant psychiatric disturbance. Notably, involuntary jaw movements were attenuated by rest and sleep, and a sensory trick (geste antagoniste) was present. The left lower facial pain was rated 7/10.

**Figure 1:**
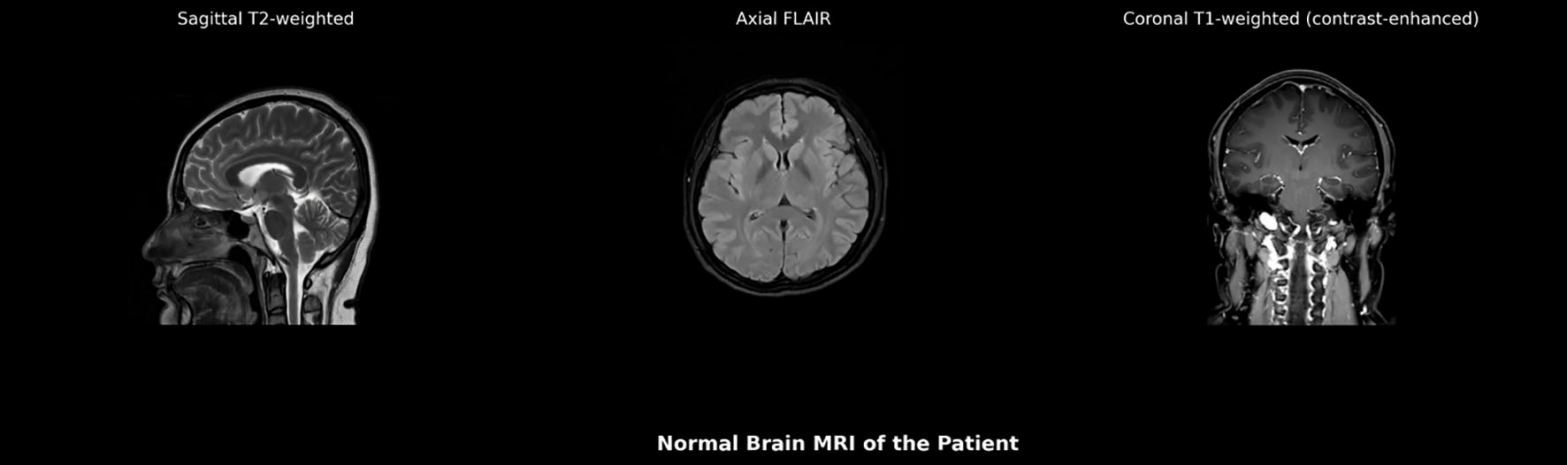
Normal brain MRI of the patient. (Left) Sagittal T2-weighted image; (Middle) Axial FLAIR image; (Right) Coronal T1-weighted contrast-enhanced image. No structural abnormalities were identified on any sequence.

Brain MRI showed no abnormalities (**Figure 1**). Cerebrospinal fluid (CSF) analysis revealed elevated immunoglobulin G (0.0168 g/L) with normal IgG synthesis rate and index. Comprehensive autoimmune encephalitis antibody screening was performed at an accredited reference laboratory (KingMed Diagnostics, China) using a cell-based assay (CBA). Anti-NMDAR IgG antibodies were positive in serum (1:32) and cerebrospinal fluid (1:1). All other neural autoantibodies were negative. Lymphocyte subset analysis showed elevated CD3+ and CD4+ T-cell percentages, consistent with active autoimmunity. Whole-exome sequencing, spinocerebellar ataxia screening, and Huntington disease gene analysis were all negative. Antinuclear antibody was positive (1:100, homogeneous pattern) and anti-histone antibody was positive.

**Figure 2:**
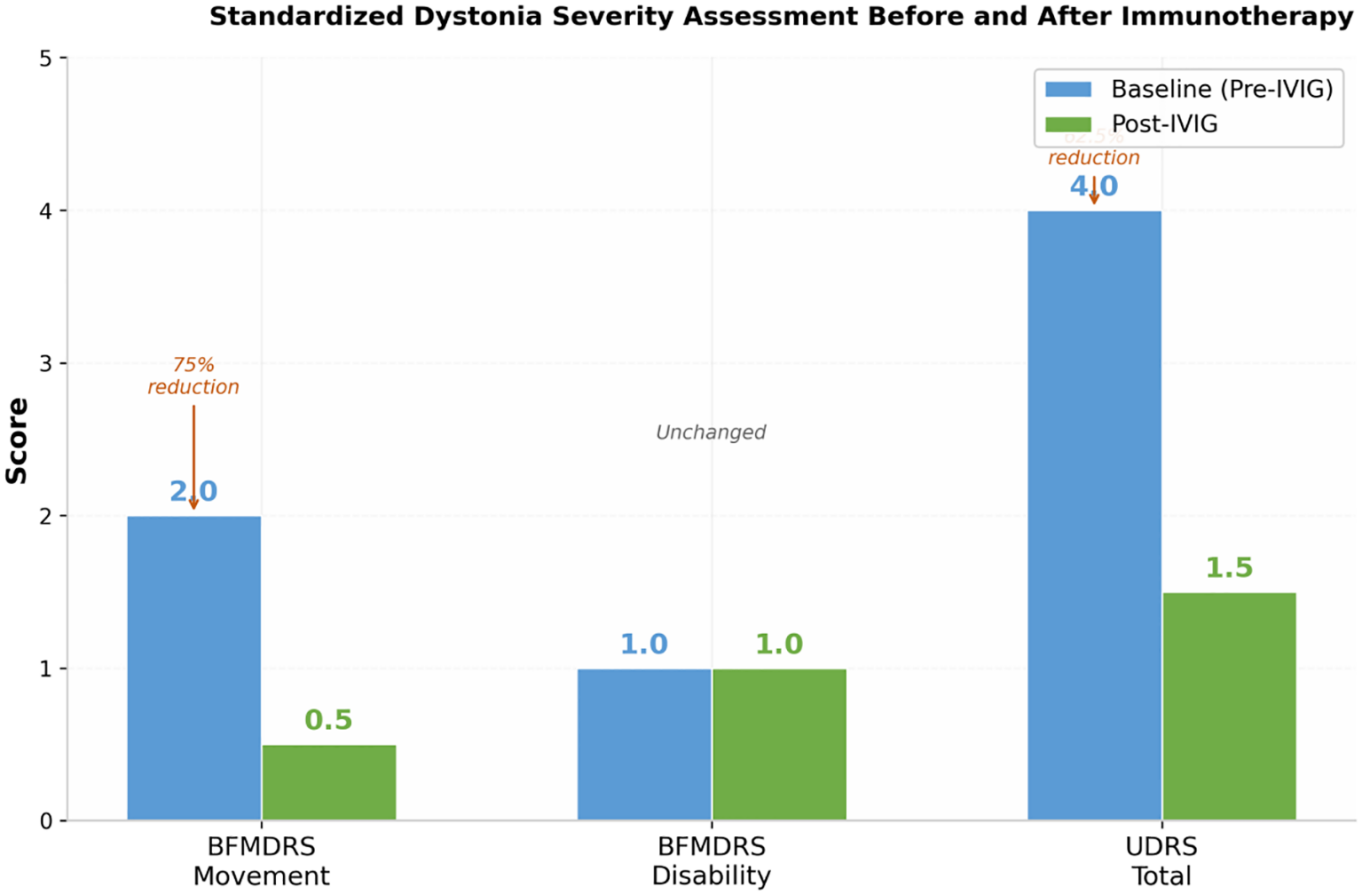
Standardized dystonia severity assessment before and after immunotherapy. BFMDRS = Burke-Fahn-Marsden Dystonia Rating Scale; UDRS = Unified Dystonia Rating Scale. The BFMDRS movement score improved from 2.0 to 0.5 (75% reduction), and the UDRS total score improved from 4.0 to 1.5 (62.5% reduction).

The Burke-Fahn-Marsden Dystonia Rating Scale (BFMDRS) movement score was 2, and the Unified Dystonia Rating Scale (UDRS) total score was 4 (**Figure 2**). Given the relatively mild and localized manifestations, together with the patient’s concerns regarding potential corticosteroid-related adverse effects, IVIG was selected as the initial first-line immunotherapy after shared decision-making. Treatment with IVIG (0.4 g/kg/day for 5 days) resulted in marked improvement (BFMDRS 0.5, UDRS 1.5). Oral prednisone was initiated post-discharge as maintenance therapy.

### 2.2 Relapse and second hospitalization (march 2023)

Five months after discharge, the patient experienced relapse following COVID-19 infection, with worsening jaw tremor, facial pain, and involuntary tongue biting. Repeat antibody testing showed elevated serum anti-NMDAR antibody titer (1:100) and CSF titer (1:100). She received IVIG for 5 days followed by high-dose methylprednisolone pulse therapy (1000 mg/day for 5 days). Mycophenolate mofetil was initiated as maintenance immunotherapy. She developed more prominent anxiety and depressive symptoms during this admission, managed with escitalopram. Epistaxis and gastrointestinal bleeding occurred, likely related to corticosteroid use, and resolved with conservative management. With maintenance immunosuppression (prednisone tapering and mycophenolate mofetil), she has remained clinically stable with sustained improvement, although mild residual focal orofacial dystonia persists.

### 2.3 Follow-up

With maintenance immunosuppression (prednisone tapering and mycophenolate mofetil), she has remained clinically stable with sustained improvement, although mild residual focal orofacial dystonia persists. At the most recent follow-up in April 2026, because mild residual focal orofacial dystonia persists despite stable disease control, deutetrabenazine (Austedo) was introduced as an empirical symptomatic treatment. The patient reported partial symptomatic improvement, although the benefit was less pronounced than that observed following immunotherapy. Botulinum toxin injection was proposed for adjunctive management of residual focal dystonia.

## 3 Discussion

We report a rare case of anti-NMDAR encephalitis presenting with a predominantly isolated orofacial motor phenotype persisting for seven months, without clinically significant psychiatric, cognitive, or seizure symptoms. This case represents a significant phenotypic expansion of the recognized clinical spectrum. Although comprehensive neuropsychological batteries were not performed, screening assessments, including MMSE, MoCA, and FAB, did not reveal clinically significant cognitive impairment. Although mild anxiety and depressive symptoms were detected on formal psychological assessment, the scores were low (HAMA 4, HAMD 2) and did not warrant psychiatric intervention. In retrospect, the mild anxiety and depressive symptoms identified on formal assessment were more likely secondary to chronic pain, involuntary tongue biting, and functional impairment than an overt neuropsychiatric manifestation of anti-NMDAR encephalitis. Furthermore, the close correlation between antibody titers and disease activity during relapse further supports the pathogenic relevance of the detected antibodies.

The relatively low initial CSF anti-NMDAR antibody titer (1:1) warrants careful interpretation. However, several observations support its clinical relevance in this case. Anti-NMDAR IgG antibodies were detected in both serum and CSF using a cell-based assay. The patient demonstrated a marked response to immunotherapy, while disease relapse was accompanied by substantial increases in antibody titers in both serum and CSF (1:100), paralleling clinical deterioration. In addition, extensive structural, genetic, and alternative autoimmune evaluations were unrevealing.

Movement disorders in anti-NMDAR encephalitis typically coexist with neuropsychiatric symptoms (9, 10). A recent analysis of 64 patients found that 76.6% presented with movement disorders, but 75.5% exhibited at least two concurrent movement abnormalities, and isolated presentations were exceedingly rare (9). Among published cases of isolated presentations, Rubio-Agusti et al. (6) described isolated hemidystonia, and Waller et al. (7) reported isolated craniocervical dystonia without initial neuropsychiatric features. Our case is unique in its exclusive orofacial presentation mimicking primary TMJ disorder for an extended duration.

Diagnostic delay in anti-NMDAR encephalitis profoundly impacts functional outcomes. A meta-analysis of 1, 550 patients demonstrated that immunotherapy initiation beyond 30 days from symptom onset independently predicts poor outcome (OR 2.7, 95% CI 1.6-4.5) (11). While 77% of patients initially present to psychiatric services (12), our patient’s seven-month delay occurred entirely within dental, oral surgery, and general neurology contexts. This pattern suggests that isolated orofacial movement disorders may pose even greater diagnostic challenges than psychiatric presentations, as they lack the behavioral changes that typically prompt autoimmune encephalitis workup.

An additional notable finding was the presence of ANA (1:100, homogeneous pattern) and anti-histone antibodies during the initial evaluation. Although these antibodies are classically associated with systemic autoimmune disorders and drug-induced lupus, the patient had no history of exposure to commonly implicated medications and did not fulfill diagnostic criteria for systemic autoimmune disease. Interestingly, ANA became negative and anti-histone antibody reactivity decreased during follow-up, paralleling clinical stabilization. These findings may reflect transient immune dysregulation accompanying anti-NMDAR encephalitis rather than a distinct autoimmune syndrome.

A temporal association was observed between the patient’s self-reported COVID-19 infection and subsequent clinical relapse, accompanied by substantial increases in antibody titers (serum 1:32 to 1:100; CSF 1:1 to 1:100). However, virological confirmation was unavailable because the infection occurred outside our institution and had clinically resolved before readmission. Therefore, although COVID-19 may have acted as a potential trigger, a causal relationship cannot be definitively established.

The persistence of mild focal dystonia despite immunological stabilization raises the possibility of residual network dysfunction or fixed motor sequelae rather than ongoing active autoimmune inflammation. This distinction may be particularly relevant when interpreting the need for continued symptomatic therapies in long-term follow-up.

The LEARN trial demonstrated that 24-month maintenance therapy with mycophenolate mofetil reduced relapse rates by 78% compared to first-line treatment alone (13). Although the LEARN trial enrolled patients with definite anti-NMDAR encephalitis rather than isolated movement-disorder phenotypes, its findings provide indirect support for maintenance immunotherapy in selected patients with recurrent disease.

This case highlights three critical lessons: First, young women presenting with subacute, treatment-refractory orofacial movement disorders deserve heightened scrutiny for autoimmune etiologies. Second, normal structural imaging should not provide false reassurance but should prompt antibody testing when clinical features are atypical. Third, maintenance immunosuppression is crucial for preventing relapse, particularly in patients with viral trigger exposure risk. Several limitations should also be acknowledged. First, comprehensive neuropsychological testing was not performed, and subtle cognitive deficits may therefore have been under-recognized despite normal screening assessments (MMSE, MoCA, and FAB). Second, objective longitudinal dystonia rating scales were not systematically collected throughout the entire follow-up period.

## 4 Patient perspective

“I spent seven months going from one doctor to another, receiving injections in my jaw and taking medications that didn’t help. No one could explain why my jaw kept moving on its own or why the pain wouldn’t go away. When I was finally diagnosed and received the immunoglobulin treatment, I felt a dramatic improvement within days. The relapse after COVID was discouraging, but the second round of treatment worked, and I have been stable since then. I hope sharing my story will help other patients with similar symptoms get diagnosed faster.”

## Data Availability

The original contributions presented in the study are included in the article/supplementary material. Further inquiries can be directed to the corresponding authors.
